# Time-Dependent Protection of CB2 Receptor Agonist in Stroke

**DOI:** 10.1371/journal.pone.0132487

**Published:** 2015-07-17

**Authors:** Seong-Jin Yu, David Reiner, Hui Shen, Kou-Jen Wu, Qing-Rong Liu, Yun Wang

**Affiliations:** 1 Center for Neuropsychiatric Research, National Health Research Institutes, Zhunan, Taiwan; 2 Neural Protective and Regeneration Section, National Institute on Drug Abuse, NIH, Baltimore, Maryland, United States of America; School of Pharmacy, Texas Tech University HSC, UNITED STATES

## Abstract

Recent studies have indicated that type 2 cannabinoid receptor (CB2R) agonists reduce neurodegeneration after brain injury through anti-inflammatory activity. The purpose of this study was to examine the time-dependent interaction of CB2R and inflammation in stroke brain. Adult male rats were subjected to right middle cerebral artery occlusion (MCAo). CB2R mRNA expression was significantly elevated >20 fold on day 2, peaked >40-fold on day 5, and normalized on day 10 post-stroke. Inflammatory markers IBA1 and TLR4 were significantly upregulated 15 fold until day 5 after MCAo. Because of the delayed upregulation of CB2R and IBA1, we next treated animals daily with CB2R agonist AM1241 or anti-inflammatory PPAR-γ agonist pioglitazone from 2 to 5 days after MCAo. Delayed treatment with pioglitazone significantly reduced abnormal neurological scores and body asymmetry as well as brain infarction in stroke animals. No behavioral improvement or reduction in brain infarction was found in animals receiving AM1241. Pioglitazone, but not AM1241, significantly reduced IBA1 expression in the stroke cortex, suggesting that delayed treatment with AM1241 failed to alter ischemia-mediated IBA-1 upregulation. In contrast, pretreatment with AM1241 significantly reduced brain infarction and neurological deficits. In conclusion, our data support a time-dependent neuroprotection of CB2 agonist in an animal model of stroke. Delayed post- treatment with PPAR-γ agonist induced behavioral recovery and microglial suppression; early treatment with CB2R agonist suppressed neurodegeneration in stroke animals.

## Introduction

Two types of cannabinoid (CB) receptors, CB1R and CB2R, have been identified. CB1R is expressed in the brain [1,2] and the periphery. CB2R is highly expressed in immune cells in peripheral tissue [3] and has been considered to be predominately a peripheral CB receptor. A few studies have reported that CB2R is also present in the CNS [4,5]. CB2R mRNA or immunoreactivity was found in cerebellar neurons, hippocampal cells [6,7], and perivascular microglia in brain [8]. Microglial activation is associated with CB2 upregulation in response inflammation in CNS [9].

Increasing evidence has supported neuroprotective roles of CB receptors in stroke [10]. Treatment with CBR agonist delta-tetrahydrocannabinol (THC) or R (+)WIN55212-2 reduced cerebral infarction, neuronal loss, and neurological deficits in experimental stroke animals [11–13]. Ischemia also modulates the expression of CB1R in brain. CB1R immunoreactivity was regionally enhanced in cortex above corpus callosum from 2 to 72 hours after proximal middle cerebral artery occlusion (MCAo) in rats [14]. On the other hand, CB1R mRNA was reduced at 5 hours in adult rats [15] or not changed at 24 hours in neonatal rats [16] after MCAo. Knocking out CB1R enhanced cerebral infarction and neurological deficits after stroke [17]. Non-selective CBR agonist R(+)-WIN 55212–2 –mediated protection was antagonized by a CB1R antagonist in stroke rats [13]. Since N-methyl d- aspartate (NMDA) caused more sever brain lesion in the CB1R knock-out mice, compared with the wild-type controls, it has been suggested that CB1R induced protection through regulating excitotoxicity [17]. Interestingly, CB1R antagonist SR141716 or LY32013 also reduced infarction or improved neurological function in stroke animals [18]. The discrepancies among these studies may attribute to the differences in animal species and stroke models used.

CB2R activation has been more consistently reported after brain injuries. CB2R –mediated protection has been attributed to the suppression of microglial activation [19,20]. The CB2R agonist 0–1966, given at 1 and 24 h after cortical contusion impact injury, reduced cerebral edema and microglial cell activation in C57BL/6 mice [21]. Overexpression of CB2R reduced microglia recruitment and dopaminergic neural degeneration in a 6-hydroxydopamine mouse model of Parkinson’s disease [22]. The protective action of CB2R has also found in animal models of stroke [23]. Systemic administration of the CB2 agonist JWH-133 at 10 minutes after a permanent MCAo suppressed the microglia marker IBA-1, neurological symptoms and infarct volume at 15 to 48 hours post stroke [15]. These data suggest that selective CB2R agonists reduced neurodegeneration associated with inflammation within 2 days post stroke. CB2R also induces neuroprotection through other mechanisms, including regulation of astroglial reactivity [24] or suppression of glutamate release [25]. As necrotic cells trigger a sterile inflammatory response [26], CB2R agonists may indirectly suppress inflammation through the reduction of cell necrosis in stroke brain

The purpose of this study was to examine the time-dependent interaction of CB2R and inflammation in stroke brain. We demonstrated that CB2R, IBA1, and TLR4 mRNA expression peaked at 5 days after MCAo. Delayed treatment with the anti-inflammatory PPAR-γ agonist pioglitazone, but not the CB2R agonist AM1241, reduced IBA1 expression in the stroke cortex and improved neurological function while pretreatment with AM1241 reduced brain damages. Our data support a time-dependent neuroprotective action of CB2R agonist for stroke.

## Materials and Methods

### Animals and MCAo

Adult male Sprague-Dawley rats, purchased from Charles River Laboratories Inc., were housed in an enriched environment by providing a toy (nylabone) or crinkle paper in their home cages with a 12 hour dark (6 pm to 6 am) and 12 hour light (6 am to 6 pm) cycle. This study was carried out in accordance with the recommendations in the Guide for the Care and Use of Laboratory Animals of the National Institutes of Health. The protocol was approved by the Committee on the Ethics of Animal Experiments of the National Health Research Institutes. All surgery was performed under anesthesia, and all efforts were made to minimize suffering. Rats were anesthetized with chloral hydrate (0.4 g/kg, i.p.). The right MCA was ligated with a 10-O suture and common carotids were clamped bilaterally by nontraumatic arterial clips to generate focal infarction in the cerebral cortex. The ligature and clips were removed after 30- or 60-min ischemia to allow reperfusion as previously described [27,28]. Core body temperature was maintained at 37°C. The average size of lesion was 190–200 mm^3^ in the right cerebral cortex at 2 days after 60 min occlusion, as determined by T2WI as described previously [27,29]. No animal died during surgery or post-stroke drug treatment. In animals receiving 30- min MCAo on day 0, brain tissue were collected for qRTPCR analysis on days 1, 2, 5 and 10 after stroke surgery.

Methods of sacrifice: Animals were anesthetized with isoflurane or chloral hydrate followed by decapitation.

### Drug administration

- Post-treatment. Animals were separated into 3 groups equal based on behavioral analysis 2 days after a 60-min MCAo. Saline, CB2R agonist AM1241 (2.5 mg/kg/d) [30], or PPAR-gamma agonist pioglitazone (1 mg/kg/d) were given intraperitoneally to the animals [31] from days 2 (after the behavioral test) to 5 after MCAo. Brain tissue was harvested for qRTPCR analysis on day 6.
- Pre-treatment. Saline or CB2R agonist AM1241 (2.5 mg/kg) was given intraperitoneally to the animals at 5-min prior to a 60-min MCAo. Brain tissue was harvested for TTC analysis two days after MCAo.

### Quantitative reverse transcription-PCR (qRT-PCR)

A 2-mm coronal section covering anterior commissure (4–6 mm from the rostral end) was harvested from each brain. Brain slices were cut into two pieces along the midline. Total RNA from left and right cortices of stroke and control rats were isolated using the TRIzol Reagent. RNA integrity numbers (RIN) were measured by Agilent 2100 Bioanalyzer (Agilent Technologies, Santa Clara, CA). The single strand cDNAs were synthesized using the Superscript III first-strand cDNA synthesis kit according to the manufacturer’s protocols (Invitrogen, Life Technologies, Carlsbad, CA). TaqMan probes (Table 1) for rat CB1R and CB2R were designed using ABI Primer Express 3.0 (Applied Biosystems, Life Technologies, Carlsbad, CA) at the splicing junctions of the rat CB1R and CB2R isoforms. The duplex PCR assays, containing both the target and endogenous control probes (Table 1), were carried out in a 7500 Fast TaqMan instrument using a default thermo-cycling program. The relative quantification calculation is according to ABI user bulletin #2. The target gene Ct value was subtracted from the endogenous control Ct value to obtain the delta-Ct value. The fold change of the left cortex was used for reference and subtracted from delta-CT of the normalized target genes in the right site cortex to obtain delta-delta-Ct values. The relative fold change is calculated using the formula 2^-(delta-delta-Ct)^. The efficiency of the target genes and the GAPDH probe amplifications was validated to be approximately equal against different concentrations of total RNA.

**Table 1:** List of qPCR TaqMan probes and primers.

Gene	TaqMan probe	Forward primer	Reverse prime
CB1R	TGAGAAGGGGTTCC	GTGCCGAGGGAGCTTCTG	GACTCAAGGTGACTGAGAAAGA
CB2R	CTGACAAATGACTCCCAGTC	CAGGACAAGGCTTCACAAGAC	GACAGGCTTTGGCTGCTTCTAC
TLR4	TGCATAGAGGTACTTCCTAAT	CCTGAGACCAGGAAGCTTGAA	TCTGATCCATGCATTGGTAGGT
GAPDH	CTCATGACCACAGTCCA	GACAACTTTGGCATCGTGGAA	CACAGTCTTCTGAGTGGCAGTGA
IBA1	Rn00574125_g1		

### Behavioral measurements

Two behavioral tests were conducted on days 2 and 6 after MCAo. All measurements were done by blinded observers.

- Body asymmetry was analyzed using an elevated body swing test [32]. Rats were examined for lateral movements/turning when their bodies were suspended 20 cm above the testing table by lifting their tails. The frequency of initial turning of the head or upper body contralateral to the ischemic side was counted in 20 consecutive trials. The maximum impairment in body asymmetry in stroke animals is 20 contralateral turns/20 trials. In non-stroke rats, the average body asymmetry is 10 contralateral turns/20 trials (i.e., the animals turn in each direction with equal frequency) [27].
- Neurological deficits were also evaluated using Bederson’s score [33]. In a postural reflex test, rats were examined for the degree of abnormal posture when suspended 20–30 cm above the testing table. They were scored according to the following criteria.
  0 Rats extend both forelimbs straight. No observable deficit.
  1 Rats keep the one forelimb to the breast and extend the other forelimb straight.
  2 Rats show decreased resistance to a lateral push in addition to the behavior in score 1 without circling.
  3 Rats twist the upper half of their body in addition to behavior in score 2.

### Primary cultures of rat cortical neurons and immunocytochemistry

Primary cultures were prepared from embryonic (E14–15) cortical tissues obtained from timed pregnant Sprague-Dawley rats. After removing the blood vessels and meninges, pooled cortices were trypsinized (0.05%; Invitrogen, Carlsbad, CA) for 20 min at room temperature. After rinsing off trypsin with prewarmed DMEM (Invitrogen), cells were dissociated by trituration, counted and plated into 96-well (5.0 x10^4^/well) cell culture plates pre-coated with polyethyleneimine (Sigma-Aldrich). The culture plating medium consisted of neurobasal medium supplemented with 2% heat-inactivated fetal bovine serum (FBS), 0.5 mM L-glutamine, 0.025mM L-glutamate and 2% B27 (Invitrogen). Cultures were maintained at 37°C in a humidified atmosphere of 5% CO_2_ and 95% air. The cultures were fed by exchanging 50% of media with feed media (neurobasal medium, Invitrogen) with 0.5 mM L-glutamate and 2% B27 with antioxidants supplement on days in vitro (DIV) 3 and 5. Cultures were fed with neurobasal media containing B27 supplement without antioxidants (Invitrogen) on DIV 7 and 10. Cultured cells were treated with glutamate (100 μM), AM1241 (10 μM), or pioglitazone (10 μM) on DIV 10 for 48 h and were fixed for microtubule-associated protein-2 (MAP2) immunostaining on DIV 12. After removing 4% PFA solution, cells were washed with phosphate-buffered saline (PBS). Fixed cells were treated with blocking solution [2% bovine serum albumin (BSA),0.1% Triton X-100 (Sigma, St. Louis, MO, USA) and 5% goat serum in PBS] for 1 hour. The cells were incubated for 1 day at 4°C with a mouse monoclonal antibody against MAP2 (1:500, Millipore, Billerica, MA, USA) and then rinsed three times with PBS. The bound primary antibody was visualized using Alexa Fluor 488 goat anti-mouse secondary (Invitrogen). Images were acquired using a monochrome camera Qi1-mc attached to the Nikon TE2000-E inverted microscope.

### Hematoxylin and eosin (H&E) and immunostaining

Animals were perfused intracardially with 4% paraformaldehyde (Sigma-Aldrich; 100 ml/minute) in 0.1 M phosphate buffer (PB; Sigma-Aldrich), pH 7.3. Brains were removed from the skull, postfixed for 18–20 hours at 4°C, rinsed with PB and sequentially transferred to 10%, 20%, and 30% sucrose solutions. Brains were then frozen on dry ice and sectioned on a cryostat to obtain coronal sections of 30 μm in thickness.

#### H&E staining

Brain sections were mounted on gelatin-coated slides and dried. Hematoxylin QS (H-3404; Vector Laboratories, Burlingame, CA, USA) was applied to each slide for 1min at room temperature. After washing, slides were incubated with 1% eosin Y solution (Vector Laboratories) for 1 min. Slides were washed again and then covered using cytoseal 60 (Thermo Scientific, Waltham, MA, USA). The area of infarction was quantified and averaged in three consecutive brain sections with a visualized anterior commissure per each animal.

#### Immunostaining

Brain sections were rinsed in phosphate buffer (PB; Sigma-Aldrich) and were blocked with 4% bovine serum albumin (BSA; Sigma-Aldrich) with 0.3% Triton X-100 (Sigma-Aldrich) in 0.1 mol/L PB. Sections were then incubated with monoclonal anti-ionized calcium-binding adapter molecule 1 (IBA1; 1:100, Chemicon, Billerica, MA, USA),monoclonal anti-CD4 (CD4; 1:100, GenWay Biotech, San Diego, CA, USA) or polyclonal anti-CD8A (CD8A; 1:100, MyBiosource Inc, San Diego, CA, USA) at 4°C overnight. Sections were rinsed in 0.1 mol/L PB and incubated in Alexa Fluor 568 secondary antibody solution (1:500; Invitrogen, Carlsbad, CA, USA). Control sections were incubated without primary antibody. Brain sections were mounted on slides and coverslipped. Confocal analysis was performed using a Nikon D-ECLIPSE 80i microscope (Melville, NY, USA) and EZ-C1 3.90 software. The optical density of IBA1 immunoreactivity, CD4, and CD8 fluorescence was quantified in three consecutive brain sections with a visualized anterior commissure in each animal. Six and three photomicrographs were taken along the peri-lesioned region and core area per brain slices; IBA1, CD4, and CD8 optical density were analyzed by NIS-Elements AR 3.2 Software (Nikon) and were averaged in each brain for statistical analysis. All histological measurements were done by blinded observers.

### Triphenyltetrazolium chloride (TTC) staining

Two days after reperfusion rats were decapitated. The brains were removed and sliced into 2.0-mm-thick sections. The brain slices were incubated in a 2% TTC solution (Sigma, St. Louis) for 15 min at room temperature and then transferred into a 4% paraformaldehyde solution for fixation. The area of infarction in each slice was measured with a digital scanner and Imagetools programs (University of Texas Health Sciences Center). The volume of infarction in each animal was obtained from the product of average slice thickness (2 mm) and the sum of infarction areas in all brain slices examined.

### Statistical analysis

Values are expressed as means ± s.e.m. Shapiro-Wilk test was used to examine the normality. Student’s t test, 1- and 2-way ANOVA tests were used for statistical analysis. ANOVA on ranks was used when the normality assumption was violated. Post-hoc Newman-Keuls test was used for all pairwise multiple comparisons. A statistically significant difference was defined as p < 0.05.

## Results

### Time-dependent activation of CB1R, CB2R, TLR4, and IBA1 after stroke

Adult rats (n = 24) were used to examine the expression of CB2R, CB1R, TLR4, and IBA1. We previously demonstrated that a 30-min distal MCAo produced minimal brain infarction in adult rats [34]. To reduce the confounding interferences of the infarcted tissue, time-dependent mRNA expression was examined in animals receiving a 30-min right MCAo. Animals were euthanized on Days 1, 2, 5 and 10 after stroke surgery (n = 6 per each time point). An additional 7 rats were used as non-stroke controls.

**Fig 1:**
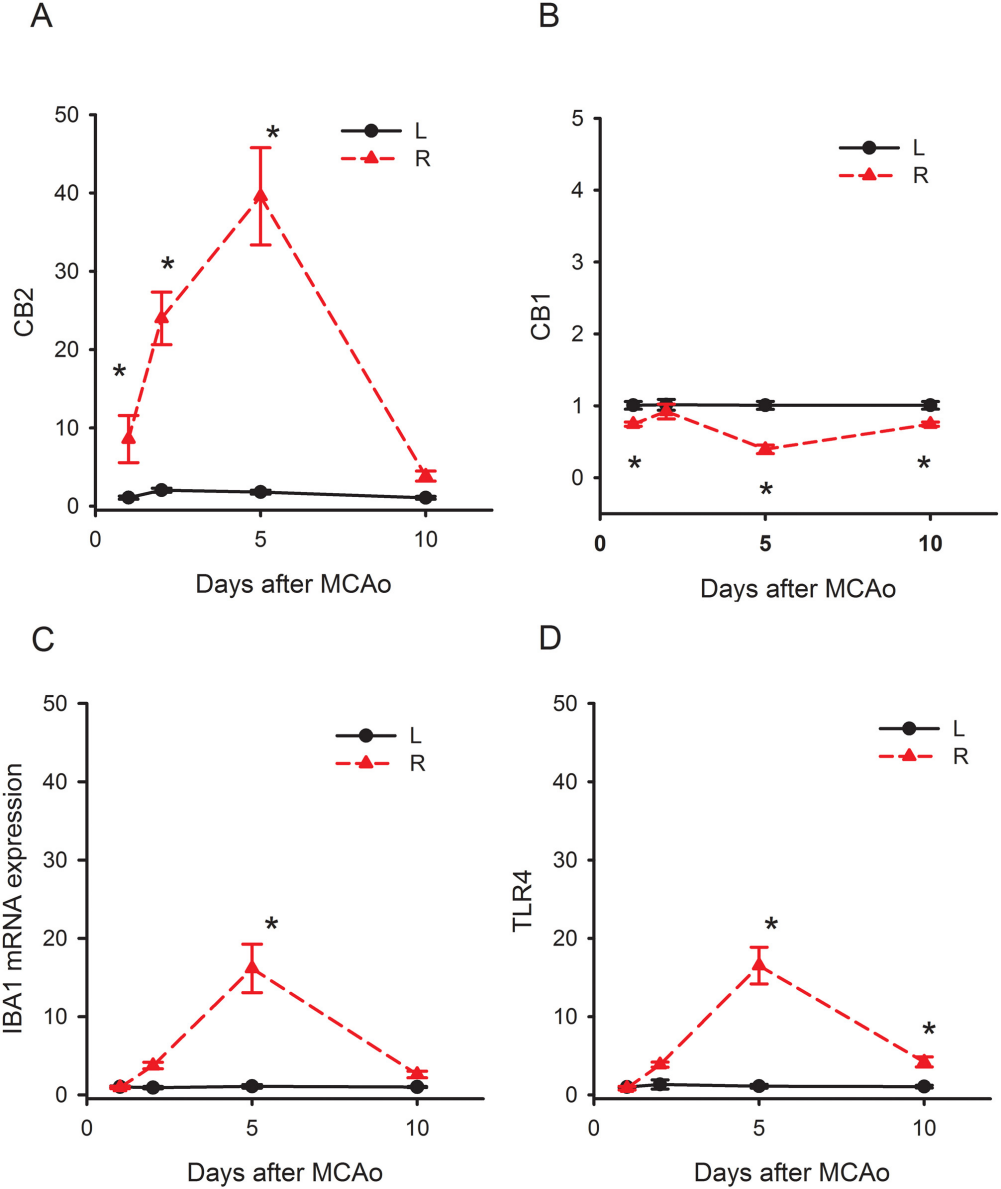
Time-dependent changes of IBA1, TLR4, CB1 and CB2 mRNA in the ischemic cortex . Animals received a 30-min right middle cerebral artery occlusion. The expression of IBA1, TLR4, CB1 and CB2 mRNA in the ischemic cortex (R, right cortex) was normalized to the non- ischemic cortex (L, left cortex). (A) IBA1 and (B) TLR4 mRNAs were significantly increased up to 15 fold on day 5 and returned to control levels on day 10 post-stroke. (C) CB1R expression was slightly (<1 fold) but significantly reduced on days 1, 5 and 10. (D) CB2R mRNA was significantly increased to 10 fold on day 1, 25 fold on day 2, and 40 fold by day 5.

We found that the expression of CB2R in the contralateral cerebral cortex of stroke rats was not different from that in the non-stroke animals (Sl Fig). The mRNA signals in the contralateral cortex were used as an internal control for comparison and were normalized to the mean mRNA level in the non-lesioned cortex collected on day 1. CB2R mRNA expression in the ischemic cortex (R, right cortex), comparing to the non- ischemic cortex (L, left cortex), was significantly increased to 10 fold on day 1, 25 folds on day 2, and 40 fold on day 5 (Fig 1A, p<0.05, two-way ANOVA on Rank + Newman-Keuls post hoc test). CB1R expression was not changed on day 2. Its expression was slightly (<1 fold) but significantly reduced on days 1, 5 and 10 (Fig 1B, p<0.05).

In contrast to CB2R, the upregulation of IBA1 and TLR4 was less prominent after stroke. The IBA1 expression was significantly enhanced on day 5 and returned to control on day 10 post-stroke (Fig 1C, p<0.05). A similar pattern of TLR4 mRNA expression was found after stroke (Fig 1D). The expression of TLR4 and IBA1 was both increased to 15 folds on day 5 in the ischemic cortex. The expression of CB2R, IBA1 or TLR4 returned to the basal level on day 10. These data suggest that CB2R, compared to IBA1 or TLR4, was upregulated much earlier (day 2 vs. day 5) and more prominent (>40 fold vs. 15 fold) by stroke.

### Delayed treatment with pioglitazone reduced neurodegeneration in stroke rats

Because the upregulation of CB2R, IBA1, and TLR4 occurred between days 2 and 5 post-stroke, we next treated animals with the CB2R agonist AM1241 (2.5 mg/kg/d) [30], the anti-inflammatory agent pioglitazone (1 mg/kg/d) [31], or vehicle from days 2 to 5. All animals received a 60-min MCAo since maximal cerebral infarction, and neurological deficits were found after 60 min distal MCAo in adult rats [34].

**Fig 2:**
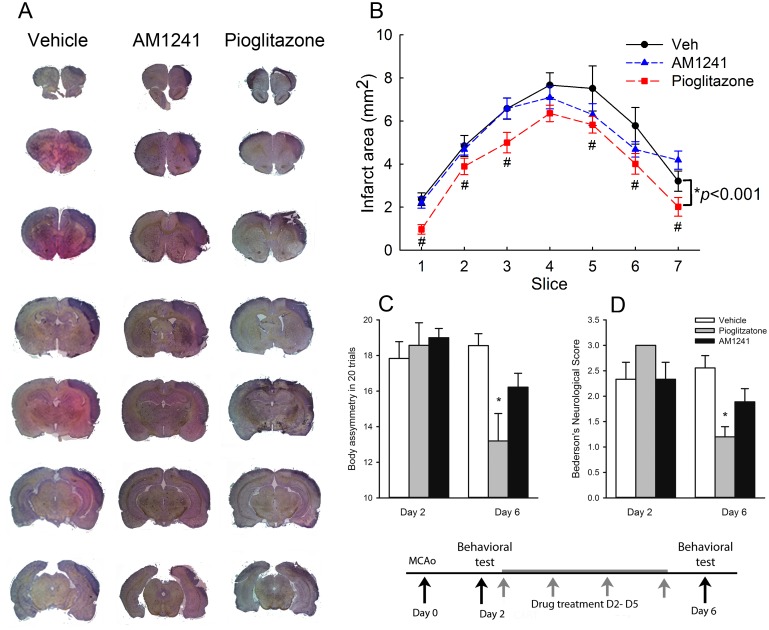
Post-stroke treatment with pioglitazone reduced brain infarction and neurological deficits in stroke rats. AM1241, pioglitazone, or vehicle was given to rats from days 2 to 5 after a 60-min MCAo. (A) Animals were sacrificed on day 6 after MCAo for H&E staining. Brain infarction was found in the lesioned side (right) cortex all stroke rats. Treatment with pioglitazone reduced the size of infarction. (B) The area of infarction in brain slices was quantified every 2 mm from the rostral end. Post treatment with pioglitazone significantly reduced brain lesion. No difference was found between vehicle and AM1241 treatment groups. (C) Body asymmetry was examined using an elevated body swing test in 20 trials per each animal on days 2 (before drug treatment) and 7 (one day after 4-day drug treatment). All animals demonstrated close to 90% body asymmetry at 2 days after MCAo. No difference was found amongst groups before treatment. After treatment with pioglitazone, there was a significant reduction in body asymmetry (p = 0.006). AM1241 did not significantly alter body asymmetry on day 6 (p = 0.158). (D) Bederson’s neurological test was carried out on days 2 and 6 after MCAo. No difference was found prior to drug treatment on day 2 (p = 0.127). Treatment with pioglitazone significantly reduced Bederson’s score on day 6 post-MCAo (p = 0.001). AM1241 did not alter Bederson’s neurological scores on day 6. (#p<0.05, two-way ANOVA; * p<0.05, one way ANOVA).

**Fig 3:**
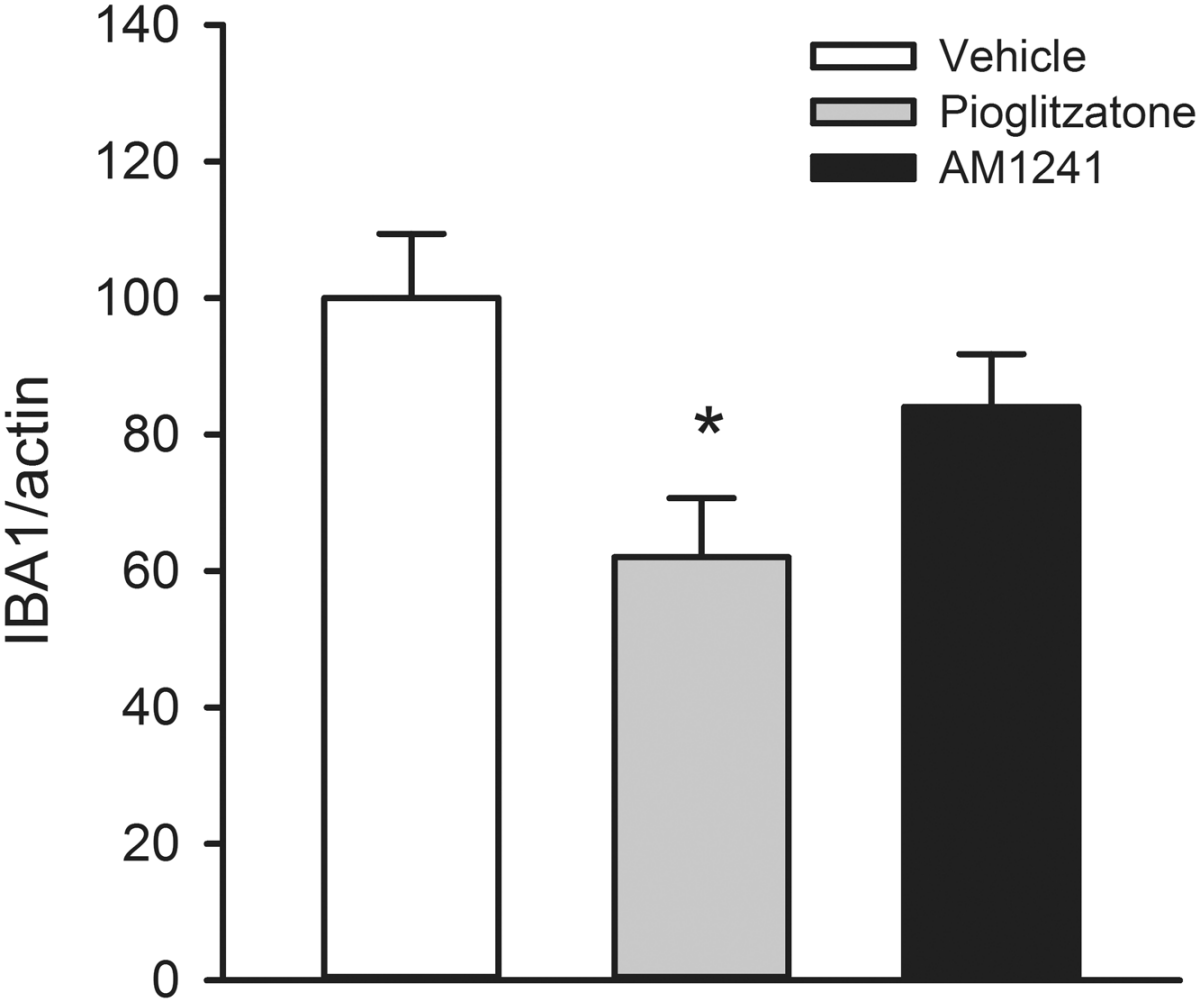
Post-treatment with pioglitazone attenuated the expression of IBA1 mRNA in lesioned cortex. Cortical tissue was collected on day 6. A significant reduction in IBA1 mRNA levels was found in rats receiving pioglitazone treatment (p = 0.023). IBA1 level was not altered by AM1241 treatment (p = 0.227).

**Fig 4:**
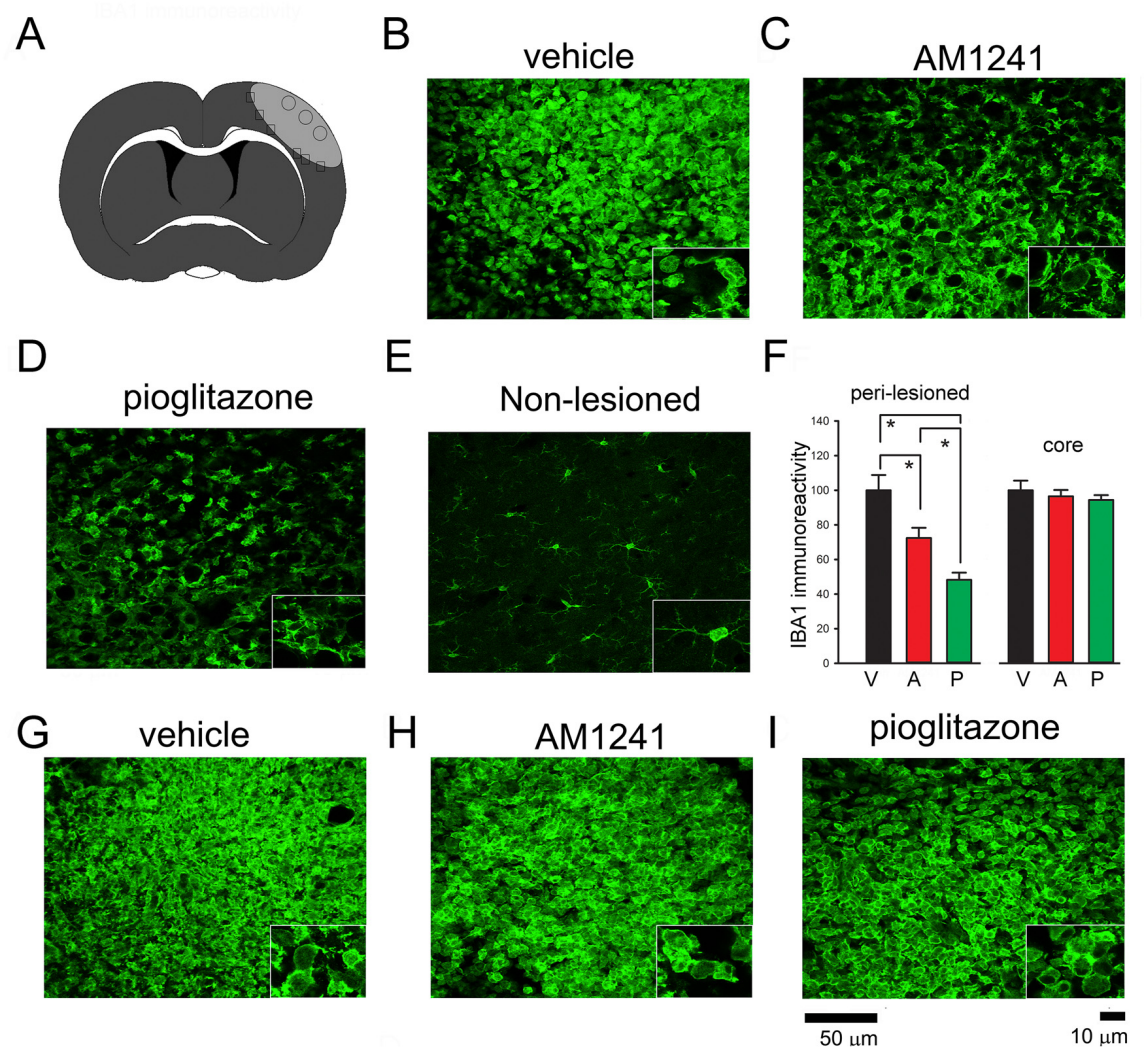
Post-treatment with pioglitazone or AM1241 reduced IBA1 immunoreactivity in the peri-lesioned area. AM1241, pioglitazone, or vehicle was given to rats from days 2 to 5 after a 60-min MCAo. Animals were sacrificed on day 6 for IBA1 immunostaining. (A) Core (circle) and peri lesioned (squares) areas in the ipsilateral cortex in each brain section with a visualized anterior commissure were used to compare IBA1 activity in all brain samples. (B-D) In the peri lesioned area, IBA1 immunoreactivity was greatly increased, comparing to (E) the non-lesioned side cortex. (B, insert) High magnification images indicated that de-ramified microglial cells in the peri lesioned region. (E, insert) In the non-lesioned side cortex, resting microglia exhibited ramified morphology. (D) Post-treatment with pioglitazone greatly reduced the IBA1 immunoreactivity and (D, insert) morphological activation of microglia in the peri-lesioned area. (C) AM1241 partially reduced IBA1 immunoreactivity. (F) The IBA1-immunofluorescence (IF) was quantified in three consecutive brain sections with a visualized anterior commissure in each animal. Averaged IBA1-IF pixel density in the peri-lesioned zone was significantly reduced by pioglitazone (p<0.001, Left panel). A significant reduction in IBA1-IFwas found in animals treated pioglitazone, compared to AM1241 (p = 0.014), suggesting that pioglitazone was more potent than AM1241 to reduce IBA1 immunoreactivity in the peri-lesioned area. (G-I) In the lesioned core, treatment with (H) AM1241 or (I) pioglitazone did not alter IBA1-IF. (F, right panel) Averaged IBA1-IF pixel density in the core was not affected by AM1241 or pioglitazone.

**Fig 5:**
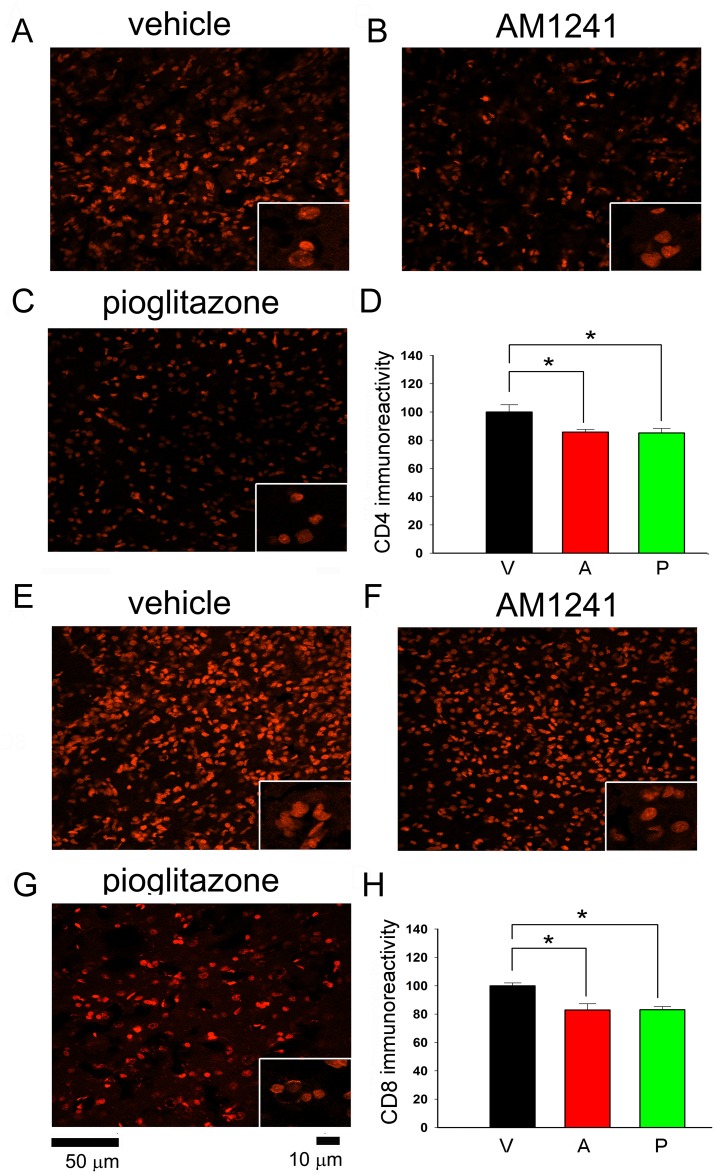
Post-treatment with pioglitazone or AM1241 reduced CD4 and CD8 immunoreactivity in the peri-lesioned area. (A-C) CD4 and (E-G) CD8 cells were found in the peri-lesioned cortex on day 6 after MCAo. Post-treatment with AM1241 or pioglitazone attenuated CD4 and CD8 immunoreactivity. Averaged (D) CD4 or (H) CD8 optical density in the peri-lesioned zone was significantly reduced by AM1241 or pioglitazone, compared with vehicle treatment (p<0.05, one-way ANOVA). No difference was found between pioglitazone or AM1241 treatments.

- Body asymmetry was examined in 28 stroke rats on days 2 (before drug treatment) and 6 (one day after a 4-day drug treatment). Elevated body swing test [35] was used to examine body asymmetry in 20 trials per each animal. Previous studies have shown that there is almost no body asymmetry in non-stroke rats [36].All animals demonstrated close to 90% body asymmetry at 2 days after MCAo (Fig 2C). No difference was found among groups before treatment (p = 0.726, one-way ANOVA). After treatment with pioglitazone, there was a significant reduction in body asymmetry (Fig 2C, p = 0.008, one-way ANOVA on Ranks; p = 0.006, posthoc Newman-Keuls test). In contrast, AM1241 did not significantly alter body asymmetry on day 6 (p = 0.158, Fig 2A).
- Bederson’s neurological test [33] was carried out on days 2 and 6 after MCAo in 28 rats. No difference was found prior to drug treatment on day 2 (p = 0.127), and Bederson’s score was close to 2.5. Treatment with pioglitazone significantly reduced Bederson’s score on day 6 post-MCAo (Fig 2D, p = 0.001, one-way ANOVA on Ranks; p = 0.001, posthoc Newman-Keuls test). No significant difference was found between vehicle and AM1241 treatment groups.
- Brain infarction: Additional 18 rats were sacrificed on day 6 after MCAo. Brain sections were stained by H&E. The area of infarction in brain slices was quantified every 2 mm from the rostral end (Fig 2A). Delayed post treatment with pioglitazone significantly reduced brain lesion (p<0.001, two-way ANOVA+ Newman-Keuls test, Fig 2B). No significant difference was found between vehicle and AM1241 treatment groups (p = 0.263, Fig 2B).
- IBA1 mRNA expression in stroke brains: The expression of IBA1 was examined in 16 stroke rats receiving 4-day treatment with vehicle (n = 6), pioglitazone (n = 5), or AM1241 (n = 5). Brain tissues were collected on day 6 after a 60-min MCAo. Pioglitazone significantly reduced IBA1 expression in the ischemic cortex (p = 0.023, Fig 3). Delayed post-stroke treatment with AM1241 did not significantly alter ischemia-mediated IBA1 expression (p = 0.227, Fig 3).IBA1, CD4, and CD8 immunoreactivity:
- IBA1 immunoreactivity was examined in 18 stroke rats on day 6 post-stroke. Using the selective microglia marker IBA1, we found that the density of IBA-1-immunofluoresence (IF) was greatly increased in the core (Fig 4G) and peri-lesioned area (Fig 4B) in the ischemic cortex, as compared to the corresponding sites in the non-lesioned side cortex (Fig 4E). High magnification images indicated that resting microglia exhibited ramified morphology in the non-lesioned side cortex (Fig 4E, insert) while de-ramified or amoeboid microglial cells were found in the lesion core (Fig 4G, insert) or peri-lesioned area (Fig 4B, insert) in animals receiving vehicle. Post-treatment with AM1241 or pioglitazone did not alter IBA1-IF in the lesioned core (Fig 4H, 4I and 4F). In the peri-lesioned area, pioglitazone significantly reduced the IBA1-IF as well as morphological activation of microglia in the peri-lesioned area (Fig 4D & 4F, insert). AM1241, less potent than pioglitazone, also reduced IBA1-IF in this area (Fig 4C). IBA1-IF pixel density in the peri-lesioned zone was further analyzed in 18 rats. Pioglitazone, compared to vehicle, significantly reduced the averaged IBA1-IF (p<0.001, one way ANOVA + Newman-Keuls test; Fig 4F, Left). A significant reduction in IBA1-IF was found in animals treated pioglitazone, compared to AM1241 (p = 0.014, one-way ANOVA + Newman-Keuls test; Fig 4F, Left), suggesting that pioglitazone was more potent than AM1241 to reduce IBA1-IF in peri-lesioned area.
- CD4 and CD8 immunoreactivity was examined in 18 rats on day 6.CD4 and CD8-IF pixel density was greatly enhanced in the core of lesioned cortex; AM1241 or pioglitazone did not alter CD4 and CD8 immunoreactivity in this region (data not shown). Post-treatment with pioglitazone or AM1241 significantly reduced the CD4- and CD8-IF in the peri-lesioned area in 18 animals studied (CD4: Fig 5A-5D; CD8 Fig 5E-5H, p<0.05, one-way ANOVA). No difference was found among groups before treatments.

### Pretreatment with CB2 agonist AM1241 reduced glutamate–mediated neurodegeneration in primary cortical neurons

**Fig 6:**
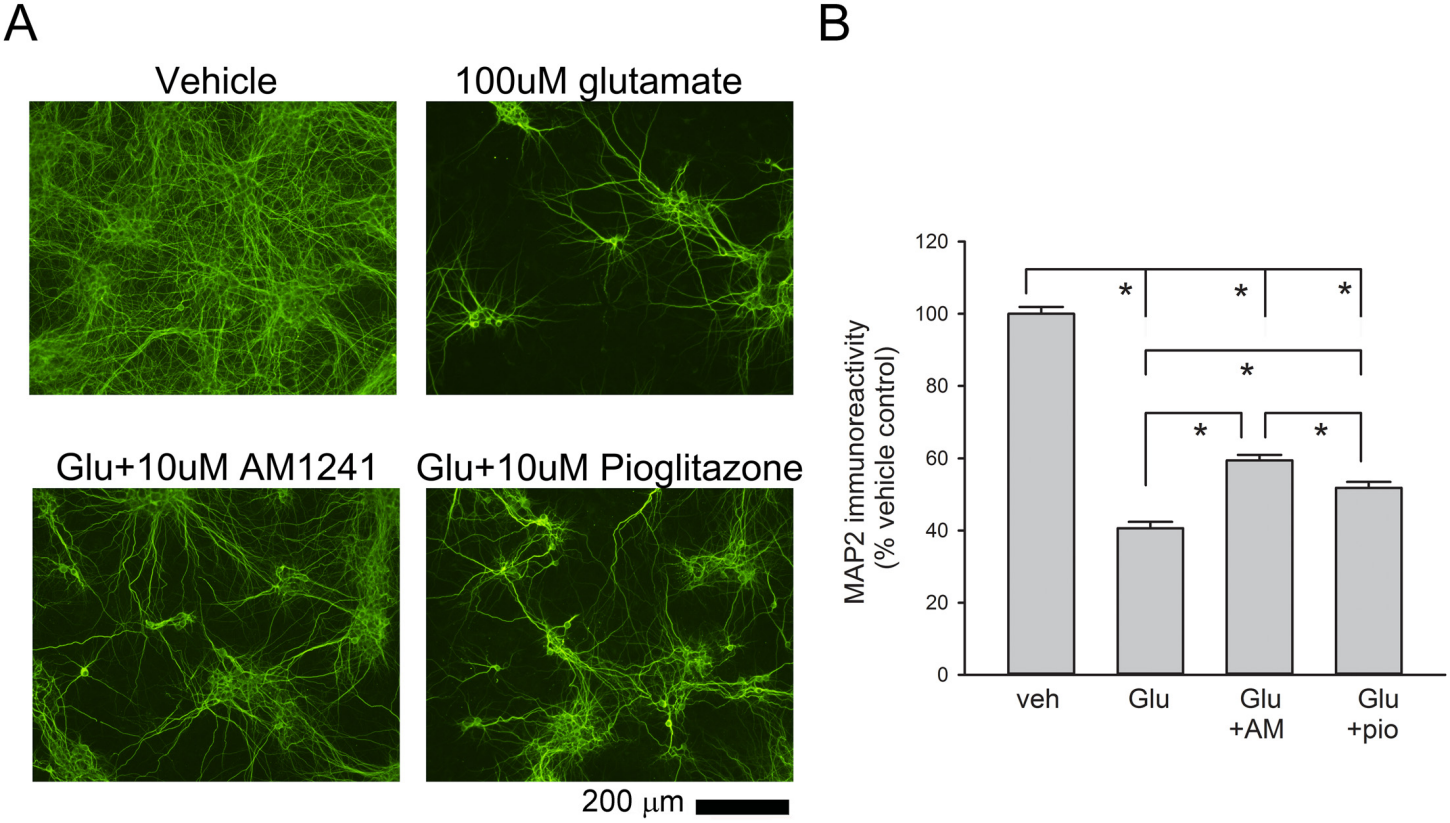
Pretreatment with AM1241 or pioglitazone preserved MAP2-immunoreactivity following exposure to glutamate in neuronal culture. (A) Photomicrographs of MAP2+ neurites in primary cortical cultures. MAP2-immunofluorescence was greatly diminished after exposure to glutamate. Pretreatment with AM1241 or pioglitazone attenuated the glutamate-related loss of MAP2 immunoreactivity. (B) Quantitation of MAP2-immunofluorescent pixel density. Glutamate significantly reduced MAP2- immunofluorescence pixel density in cortical cultures. AM1241 was more potent than pioglitazone to antagonize glutamate-mediated loss of MAP2 immunoreactivity. (*p<0.05, one-way ANOVA).

Primary cortical neuronal cultures (DIV10) were treated with AM1241 (10 ·M), pioglitazone (10 ·M), or vehicle at 5 min prior to glutamate (100 ·M). Two days later, cultured cells were fixed for MAP2 immunostaining. Treatment with glutamate significantly reduced MAP2-IF, which was antagonized by AM1241 or pioglitazone (p<0.001, one way ANOVA + Newman-Keuls test, Fig 6). AM1241 was more potent than pioglitazone to antagonize glutamate-mediated loss of MAP2-IF. (p = 0.003, one-way ANOVA + Newman-Keuls test, Fig 6B).

### Pretreatment with CB2 agonist AM1241 reduced behavioral deficits and brain infarction in stroke rats

**Fig 7:**
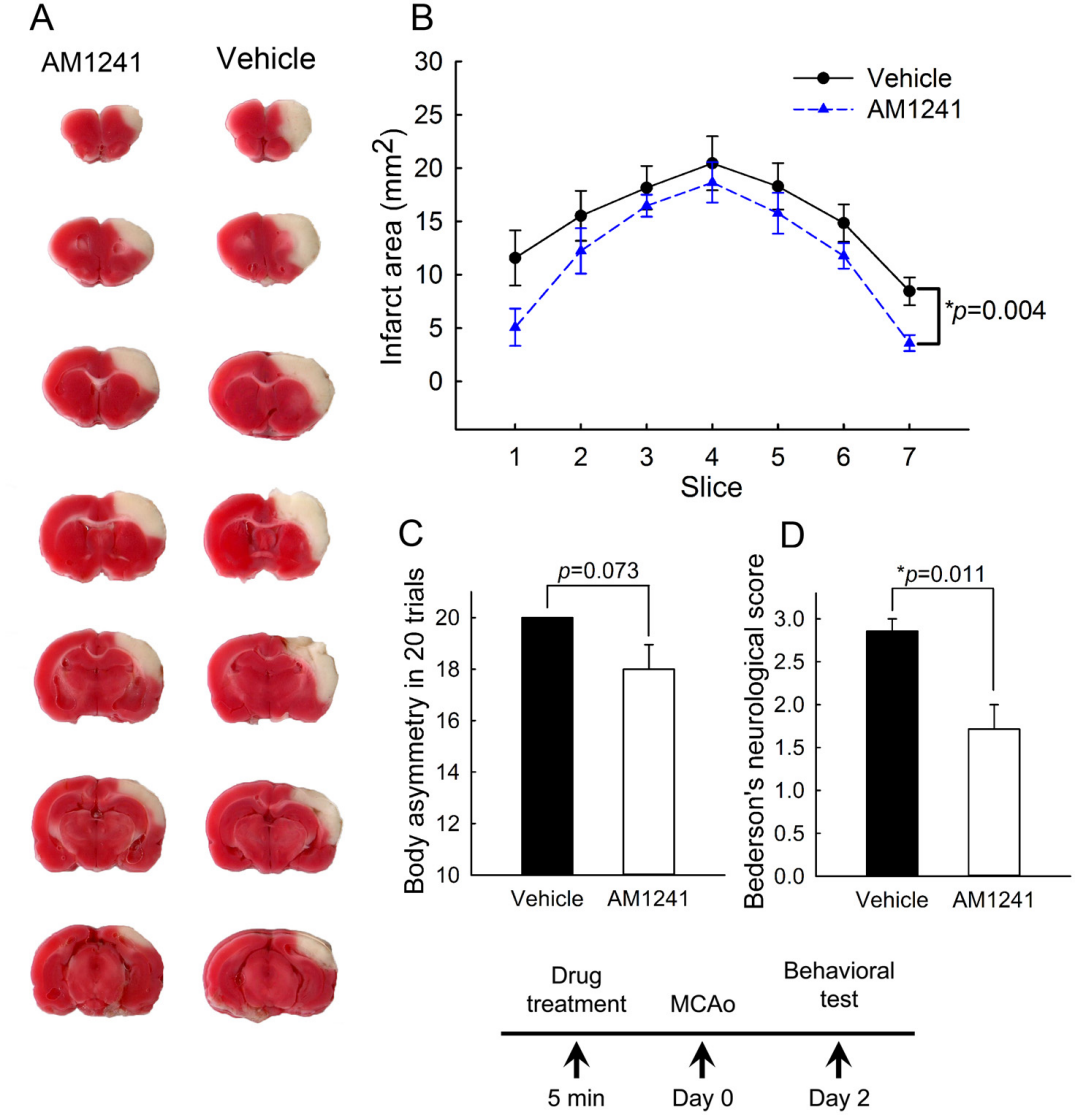
Pretreatment with CB2 agonist AM1241 reduced behavioral deficits and brain infarction in stroke rats. Rats were treated with AM1241 (2.5 mg/kg, n = 7) or vehicle (n = 7) at 5-min prior to MCAo. (A) Representing TTC histology taken at day 2 after MCAo indicates that the size of the lesion was reduced in an animal receiving AM1241. (B) The area of infarction in brain slices was quantified every 2 mm from the rostral end. AM1241 significantly reduced brain lesion (p<0.001, two-way ANOVA+ Newman-Keuls test). (C, D) Neurological tests were evaluated on day 2 post-stroke. Pretreatment with AM1241 significantly reduced (D) Bederson’s neurological scores (p = 0.011) and (C) marginally mitigated body asymmetry (p = 0.073).

Rats were treated with AM1241 (2.5 mg/kg, n = 7) or vehicle (n = 7) at 5-min prior to MCAo. Neurological tests were evaluated on day 2 post-stroke. Pretreatment with AM1241 significantly reduced Bederson’s neurological scores (p = 0.011, t-test, Fig 7D). There was a trend toward significance in the reduction of body asymmetry by AM1241 (p = 0.073, t-test, Fig 7C). All animals (n = 14) were sacrificed after the behavioral test for TTC staining. The area of infarction in brain slices was quantified every 2 mm from the rostral end (Fig 7A). Pretreatment with AM1241 significantly reduced brain infarction (p<0.001, two-way ANOVA+ Newman-Keuls test, Fig 7B).

## Discussion

In this study, we reported that post-treatment with pioglitazone, but not AM1241, from days 2 to 5 reduced brain infarction and attenuated neurological deficits in stroke animals. In contrast, pre-treatment with AM1241 reduced brain damages. Our data support a differential protective reaction of anti-inflammatory agent pioglitazone and CB2R agonist AM1241 in stroke brain. The effectiveness of protection of CB2R agonist is affected by the timing of treatment.

TLR4 is a toll-like receptor (TLR), which initiates innate immune responses. TLR4 is present mainly on microglia and astrocytes in the CNS after inflammation or MCAo [37,38]. Deficiency in TLR4 reduced infarction and inflammation after MCAo [32]. Although TLR4 is not present in neurons, its activation leads to neuronal death in the presence of microglia [37]. In this study, we demonstrated that the expression of and TLR4, similar to IBA1, was significantly increased on day 5 in stroke brain. Our data may support a delayed onset of inflammation at days after MCAo [19, 20].

Previous studies have shown that CB2R is mainly present in microglia [5] and activation of microglia is associated with CB2 upregulation in response to inflammation in CNS [6]. Since ischemic brain injury can induce neurodegeneration through inflammation, the expression of CB2R and the microglial marker IBA1 after ischemic brain injury were examined in this study. We demonstrated that both IBA1 and CB2R were upregulated after MCAo. The expression profiles of CB2R and IBA1 was similar that both gradually increased from day 1 to day 5 and then returned to basal level on day 10. The efficacy of CB2R expression was much higher than IBA1 after stroke. CB2R, compared to IBA1, was upregulated much earlier (day 2 vs. day 5) and more prominent (>40 fold vs. 15- fold) by stroke. Since CB2R has also been found in neurons [6,7], it may be of interest to further investigate the expression of CB2R protein in neurons and microglia in the brain following stroke with selective and reliable antibody for CB2R.

AM 1241 is a relatively specific CB2 agonist [39,40] with more than 100 fold selectivity over CB1 receptor [41]. In this study, we applied pioglitazone (1 mg/kg) or AM1241 (2.5 mg/kg) during the CB2R, TLR4 and IBA1 activation periods from 2 to 5 days after MCAo. It has been shown that pioglitazone suppressed inflammation in stroke brain without altering blood glucose level [31] and AM1241 attenuated neuroinflammation induced by repeated morphine administration at these doses [30]. We found that post-treatment with pioglitazone significantly reduced brain infarction, neurological score, and IBA1 expression, suggesting a neuroprotective effect through the suppression of inflammation. AM1241 slightly reduced CD4 and CD8 lymphocyte infiltration in the peri-lesioned area but failed to alter brain infarction, neurological deficits and IBA1 mRNA expression in stroke brain. AM1241 was also less effective than pioglitazone in reducing microglia activation in the peri-lesioned area. These data suggest that delayed post-stroke treatment with a CB2R agonist AM1241 cannot efficiently suppress microglial activation, brain damages or behavioral deficits in stroke animals. In contrast, AM1241, given at 5-min before MCAo, significantly reduced the area of infarction and neurological symptoms in stroke rats. A similar report has also indicated that CB2 agonists, given either before or 10 min after the beginning of MCAo, reduced the microglial activation, neurological symptoms, and infarct volume [15], suggesting that early treatment with CB2 agonists reduced ischemic brain injury. Taken together, these data support a time-dependent neuroprotection of CB2R agonist in stroke.

CB2R –mediated protection has been commonly associated with the suppression of microglial activation [19,20]. However, other mechanisms may also be involved. For example, CB2R agonist COR167 reduced oxygen-glucose deprivation (OGD)-promoted glutamate release in rat brain cortical slices [25]. CB2R agonist, JWH015 significantly protected human retinal pigment epithelial cells from oxidative damage [42]. CB2R antagonist AM630 abolished the antioxidant and cytoprotective effect of β-Caryophyllene against glutamate excitotoxicity in C6 glioma cells [43]. Pretreatment with CB2 agonist O-1966 attenuated adhesion molecule expression and blood-brain barrier disruption during cerebral ischemic and reperfusion injury [44]. In our animal study, we found that AM1241 protected against ischemic brain damage before the upregulation of IBA1 or TLR4. In the in vitro study, pre-treatment with AM1241 was more potent than pioglitazone in reducing glutamate-mediated neuronal damages in primary cortical culture, which contained minimal microglia. These data suggest that AM1241 pretreatment–mediated neuroprotection may not exclusively involve microglial suppression. As inflammation can be triggered by cell necrosis [26], CB2R agonists may also indirectly suppress inflammation through the reduction of cell necrosis in stroke brain.

In summary, our data support a time-dependent neuroprotection of CB2R agonist and upregulation of CB2R in an animal model of stroke. Delayed post-stroke treatment with a CB2R agonist was insufficient to suppress brain infarction or induce behavioral recovery while early treatment with CB2R agonist reduced ischemic brain injury. Elucidating the cellular distribution and physiological function of CB2R upregulation at days after MCAo will be important to further characterize the complex roles of CB2R in stroke brain.

## Supporting Information

S1 Fig CB2 mRNA expression in the ischemic (right) and non-ischemic (left) side cortex of stroke rats as well as in the cerebral cortex of naïve rats. Stroke animals received a 30-min right MCAo. CB2R mRNA was significantly increased to 25 fold on day 2, and 40 fold by day 5. No difference was found between brain tissue collected from the naïve animals and left cortex from the stroke rats.
(DOCX)

## References

[1] Rinaldi-Carmona M, Pialot F, Congy C, Redon E, Barth F, Bachy A, et al (1996) Characterization and distribution of binding sites for [3H]-SR 141716A, a selective brain (CB1) cannabinoid receptor antagonist, in rodent brain. Life Sci 58: 1239–1247. 0024320596000859 [pii]. 8614277 10.1016/0024-3205(96)00085-9

[2] Gatley SJ, Gifford AN, Volkow ND, Lan R, Makriyannis A (1996) 123I-labeled AM251: a radioiodinated ligand which binds in vivo to mouse brain cannabinoid CB1 receptors. Eur J Pharmacol 307: 331–338. 0014299996002798 [pii]. 8836622 10.1016/0014-2999(96)00279-8

[3] Lynn AB, Herkenham M (1994) Localization of cannabinoid receptors and nonsaturable high-density cannabinoid binding sites in peripheral tissues of the rat: implications for receptor-mediated immune modulation by cannabinoids. J Pharmacol Exp Ther 268: 1612–1623. 8138973

[4] Brusco A, Tagliaferro PA, Saez T, Onaivi ES (2008) Ultrastructural localization of neuronal brain CB2 cannabinoid receptors. Ann N Y Acad Sci 1139: 450–457. NYAS1139037 [pii]; 10.1196/annals.1432.037 18991892

[5] Xi ZX, Peng XQ, Li X, Song R, Zhang HY, Liu QR, et al (2011) Brain cannabinoid CB(2) receptors modulate cocaine's actions in mice. Nat Neurosci 14: 1160–1166. nn.2874 [pii]; 10.1038/nn.2874 21785434 PMC3164946

[6] Gong JP, Onaivi ES, Ishiguro H, Liu QR, Tagliaferro PA, Brusco A, et al (2006) Cannabinoid CB2 receptors: immunohistochemical localization in rat brain. Brain Res 1071: 10–23. S0006-8993(05)01623-9 [pii]; 10.1016/j.brainres.2005.11.035 16472786

[7] Onaivi ES, Ishiguro H, Gong JP, Patel S, Perchuk A, Meozzi PA, et al (2006) Discovery of the presence and functional expression of cannabinoid CB2 receptors in brain. Ann N Y Acad Sci 1074: 514–536. 1074/1/514 [pii]; 10.1196/annals.1369.052 17105950

[8] Nunez E, Benito C, Pazos MR, Barbachano A, Fajardo O, Gonzalez S, et al (2004) Cannabinoid CB2 receptors are expressed by perivascular microglial cells in the human brain: an immunohistochemical study. Synapse 53: 208–213. 10.1002/syn.20050 15266552

[9] Maresz K, Carrier EJ, Ponomarev ED, Hillard CJ, Dittel BN (2005) Modulation of the cannabinoid CB2 receptor in microglial cells in response to inflammatory stimuli. J Neurochem 95: 437–445. JNC3380 [pii]; 10.1111/j.1471-4159.2005.03380.x 16086683

[10] Capettini LS, Savergnini SQ, da Silva RF, Stergiopulos N, Santos RA, Mach F, et al (2012) Update on the role of cannabinoid receptors after ischemic stroke. Mediators Inflamm 2012: 824093 10.1155/2012/824093 22577257 PMC3337695

[11] Sidney S (2002) Cardiovascular consequences of marijuana use. J Clin Pharmacol 42: 64S–70S. 12412838 10.1002/j.1552-4604.2002.tb06005.x

[12] Hayakawa K, Mishima K, Nozako M, Hazekawa M, Ogata A, Fujioka M, et al (2007) Delta9-tetrahydrocannabinol (Delta9-THC) prevents cerebral infarction via hypothalamic-independent hypothermia. Life Sci 80: 1466–1471. S0024-3205(07)00063-X [pii]; 10.1016/j.lfs.2007.01.014 17289082

[13] Nagayama T, Sinor AD, Simon RP, Chen J, Graham SH, Jin K, et al (1999) Cannabinoids and neuroprotection in global and focal cerebral ischemia and in neuronal cultures. J Neurosci 19: 2987–2995. 10191316 10.1523/JNEUROSCI.19-08-02987.1999 PMC6782289

[14] Jin KL, Mao XO, Goldsmith PC, Greenberg DA (2000) CB1 cannabinoid receptor induction in experimental stroke. Ann Neurol 48: 257–261. 10939579

[15] Zarruk JG, Fernandez-Lopez D, Garcia-Yebenes I, Garcia-Gutierrez MS, Vivancos J, Nombela F, et al (2012) Cannabinoid type 2 receptor activation downregulates stroke-induced classic and alternative brain macrophage/microglial activation concomitant to neuroprotection. Stroke 43: 211–219. STROKEAHA.111.631044 [pii]; 10.1161/STROKEAHA.111.631044 22020035

[16] Fernandez-Lopez D, Faustino J, Derugin N, Wendland M, Lizasoain I, Moro MA, et al (2012) Reduced infarct size and accumulation of microglia in rats treated with WIN 55,212–2 after neonatal stroke. Neuroscience 207: 307–315. S0306-4522(12)00030-9 [pii]; 10.1016/j.neuroscience.2012.01.008 22285309 PMC3446851

[17] Parmentier-Batteur S, Jin K, Mao XO, Xie L, Greenberg DA (2002) Increased severity of stroke in CB1 cannabinoid receptor knock-out mice. J Neurosci 22: 9771–9775. 22/22/9771 [pii]. 12427832 10.1523/JNEUROSCI.22-22-09771.2002 PMC6757835

[18] Muthian S, Rademacher DJ, Roelke CT, Gross GJ, Hillard CJ (2004) Anandamide content is increased and CB1 cannabinoid receptor blockade is protective during transient, focal cerebral ischemia. Neuroscience 129: 743–750. S0306-4522(04)00768-7 [pii]; 10.1016/j.neuroscience.2004.08.044 15541895

[19] Maresz K, Carrier EJ, Ponomarev ED, Hillard CJ, Dittel BN (2005) Modulation of the cannabinoid CB2 receptor in microglial cells in response to inflammatory stimuli. J Neurochem 95: 437–445. JNC3380 [pii]; 10.1111/j.1471-4159.2005.03380.x 16086683

[20] Palazuelos J, Aguado T, Pazos MR, Julien B, Carrasco C, Resel E, et al (2009) Microglial CB2 cannabinoid receptors are neuroprotective in Huntington's disease excitotoxicity. Brain 132: 3152–3164. awp239 [pii]; 10.1093/brain/awp239 19805493

[21] Elliott MB, Tuma RF, Amenta PS, Barbe MF, Jallo JI (2011) Acute effects of a selective cannabinoid-2 receptor agonist on neuroinflammation in a model of traumatic brain injury. J Neurotrauma 28: 973–981. 10.1089/neu.2010.1672 21332427

[22] Ternianov A, Perez-Ortiz JM, Solesio ME, Garcia-Gutierrez MS, Ortega-Alvaro A, Navarrete F, et al (2012) Overexpression of CB2 cannabinoid receptors results in neuroprotection against behavioral and neurochemical alterations induced by intracaudate administration of 6-hydroxydopamine. Neurobiol Aging 33: 421–16. S0197-4580(10)00392-1 [pii]; 10.1016/j.neurobiolaging.2010.09.012 20980074

[23] Zhang M, Martin BR, Adler MW, Razdan RK, Jallo JI, Tuma RF (2007) Cannabinoid CB(2) receptor activation decreases cerebral infarction in a mouse focal ischemia/reperfusion model. J Cereb Blood Flow Metab 27: 1387–1396. 9600447 [pii]; 10.1038/sj.jcbfm.9600447 17245417 PMC2637559

[24] Garcia-Ovejero D, Arevalo-Martin A, Petrosino S, Docagne F, Hagen C, Bisogno T, et al (2009) The endocannabinoid system is modulated in response to spinal cord injury in rats. Neurobiol Dis 33: 57–71. S0969-9961(08)00225-8 [pii]; 10.1016/j.nbd.2008.09.015 18930143

[25] Contartese A, Valoti M, Corelli F, Pasquini S, Mugnaini C, Pessina F, et al (2012) A novel CB2 agonist, COR167, potently protects rat brain cortical slices against OGD and reperfusion injury. Pharmacol Res 66: 555–563. S1043-6618(12)00163-6 [pii]; 10.1016/j.phrs.2012.08.003 23036353

[26] Iyer SS, Pulskens WP, Sadler JJ, Butter LM, Teske GJ, Ulland TK, et al (2009) Necrotic cells trigger a sterile inflammatory response through the Nlrp3 inflammasome. Proc Natl Acad Sci U S A 106: 20388–20393. 0908698106 [pii]; 10.1073/pnas.0908698106 19918053 PMC2787135

[27] Luo Y, Shen H, Liu HS, Yu SJ, Reiner DJ, Harvey BK, et al (2013) CART peptide induces neuroregeneration in stroke rats. J Cereb Blood Flow Metab 33: 300–310. jcbfm2012172 [pii]; 10.1038/jcbfm.2012.172 23211962 PMC3564201

[28] Luo Y, Kuo CC, Shen H, Chou J, Greig NH, Hoffer BJ, et al (2009) Delayed treatment with a p53 inhibitor enhances recovery in stroke brain. Ann Neurol 65: 520–530. 10.1002/ana.21592 19475672 PMC2690614

[29] Liu HS, Shen H, Harvey BK, Castillo P, Lu H, Yang Y, et al (2011) Post-treatment with amphetamine enhances reinnervation of the ipsilateral side cortex in stroke rats. Neuroimage 56: 280–289. S1053-8119(11)00205-9 [pii]; 10.1016/j.neuroimage.2011.02.049 21349337 PMC3070415

[30] Tumati S, Largent-Milnes TM, Keresztes A, Ren J, Roeske WR, Vanderah TW, et al (2012) Repeated morphine treatment-mediated hyperalgesia, allodynia and spinal glial activation are blocked by co-administration of a selective cannabinoid receptor type-2 agonist. J Neuroimmunol 244: 23–31. S0165-5728(11)00373-0 [pii]; 10.1016/j.jneuroim.2011.12.021 22285397 PMC3298577

[31] Sundararajan S, Gamboa JL, Victor NA, Wanderi EW, Lust WD, Landreth GE (2005) Peroxisome proliferator-activated receptor-gamma ligands reduce inflammation and infarction size in transient focal ischemia. Neuroscience 130: 685–696. S0306-4522(04)00957-1 [pii]; 10.1016/j.neuroscience.2004.10.021 15590152

[32] Borlongan CV, Sanberg PR (1995) Elevated body swing test: a new behavioral parameter for rats with 6-hydroxydopamine-induced hemiparkinsonism. J Neurosci 15: 5372–5378. 7623159 10.1523/JNEUROSCI.15-07-05372.1995 PMC6577895

[33] Bederson JB, Pitts LH, Tsuji M, Nishimura MC, Davis RL, Bartkowski H (1986) Rat middle cerebral artery occlusion: evaluation of the model and development of a neurologic examination. Stroke 17: 472–476. 3715945 10.1161/01.str.17.3.472

[34] Shen H, Wang Y (2010) Correlation of locomotor activity and brain infarction in rats with transient focal ischemia. J Neurosci Methods 182: 150–154. 10.1016/j.jneumeth.2009.11.008 PMC2975960 19917312

[35] Borlongan CV, Hida H, Nishino H (1998) Early assessment of motor dysfunctions aids in successful occlusion of the middle cerebral artery. Neuroreport 9: 3615–3621. 9858369 10.1097/00001756-199811160-00012

[36] Chang CF, Morales M, Chou J, Chen HL, Hoffer BJ, Wang Y (2002) Bone morphogenetic proteins are involved in fetal kidney tissue transplantation-induced neuroprotection in stroke rats. Neuropharmacology 43: 418–426. 12243771 10.1016/s0028-3908(02)00092-8

[37] Lehnardt S, Massillon L, Follett P, Jensen FE, Ratan R, Rosenberg PA, et al (2003) Activation of innate immunity in the CNS triggers neurodegeneration through a Toll-like receptor 4-dependent pathway. Proc Natl Acad Sci U S A 100: 8514–8519. 10.1073/pnas.1432609100 1432609100 [pii]. 12824464 PMC166260

[38] Caso JR, Pradillo JM, Hurtado O, Lorenzo P, Moro MA, Lizasoain I (2007) Toll-like receptor 4 is involved in brain damage and inflammation after experimental stroke. Circulation 115: 1599–1608. CIRCULATIONAHA.106.603431 [pii]; 10.1161/CIRCULATIONAHA.106.603431 17372179

[39] Bingham B, Jones PG, Uveges AJ, Kotnis S, Lu P, Smith VA, et al (2007) Species-specific in vitro pharmacological effects of the cannabinoid receptor 2 (CB2) selective ligand AM1241 and its resolved enantiomers. Br J Pharmacol 151: 1061–1070. 0707303 [pii]; 10.1038/sj.bjp.0707303 17549048 PMC2042933

[40] Wilkerson JL, Gentry KR, Dengler EC, Wallace JA, Kerwin AA, Kuhn MN, et al (2012) Immunofluorescent spectral analysis reveals the intrathecal cannabinoid agonist, AM1241, produces spinal anti-inflammatory cytokine responses in neuropathic rats exhibiting relief from allodynia. Brain Behav 2: 155–177. 10.1002/brb3.44 22574283 PMC3345359

[41] Malan TP Jr., Ibrahim MM, Deng H, Liu Q, Mata HP, Vanderah T, et al (2001) CB2 cannabinoid receptor-mediated peripheral antinociception. Pain 93: 239–245. S0304395901003219 [pii]. 11514083 10.1016/S0304-3959(01)00321-9

[42] Wei Y, Wang X, Wang L (2009) Presence and regulation of cannabinoid receptors in human retinal pigment epithelial cells. Mol Vis 15: 1243–1251. 132 [pii]. 19547718 PMC2697670

[43] Assis LC, Straliotto MR, Engel D, Hort MA, Dutra RC, de Bem AF (2014) beta-Caryophyllene protects the C6 glioma cells against glutamate-induced excitotoxicity through the Nrf2 pathway. Neuroscience 279: 220–231. S0306-4522(14)00721-0 [pii]; 10.1016/j.neuroscience.2014.08.043 25194788

[44] Zhang M, Adler MW, Abood ME, Ganea D, Jallo J, Tuma RF (2009) CB2 receptor activation attenuates microcirculatory dysfunction during cerebral ischemic/reperfusion injury. Microvasc Res 78: 86–94. S0026-2862(09)00087-9 [pii]; 10.1016/j.mvr.2009.03.005 19332079 PMC3319431

