# Supplementary material for: Time-Dependent Protection of CB2 Receptor Agonist in Stroke
Source: PLoS One. 2015 Jul 17;10(7):e0132487. doi: 10.1371/journal.pone.0132487 (PMC4505877; doi:10.1371/journal.pone.0132487)
Supplement: S1 Fig — Stroke animals received a 30-min right MCAo. CB2R mRNA was significantly increased to 25 fold on day 2, and 40 fold by day 5. No difference was found between brain tissue collected from the naïve animals and left cortex from the stroke rats. (DOCX) [file pone.0132487.s001.docx]

Supplemental Figure 1: CB2 mRNA expression in the ischemic (right) and non-ischemic (left) side cortex of stroke rats as well as in the cerebral cortex of naïve rats. Stroke animals received a 30-min right MCAo. CB2R mRNA was significantly increased to 25 fold on day 2, and 40 fold by day 5. No difference was found between brain tissue collected from the naïve animals and left cortex from the stroke rats.
